# The Effects of Silver and Potassium Iodide on Honey Bee (*Apis mellifera*) Learning

**DOI:** 10.3390/insects16111157

**Published:** 2025-11-12

**Authors:** Riley J. Wincheski, Trey Mathews, Harrington Wells, Robert J. Sheaff, Lily A. Anderson, James W. Grice, Charles I. Abramson

**Affiliations:** 1 Applied Research and Analysis Company LLC, Richmond, VA 23233, USA; rwincheski@aracscience.com; 2 Laboratory of Comparative Psychology and Behavioral Biology, Oklahoma State University, Stillwater, OK 74078, USA; lily.anderson@okstate.edu; 3 Department of Biological Science, University of Tulsa, Tulsa, OK 74104, USA; tam7302@utulsa.edu (T.M.); harrington-wells@utulsa.edu (H.W.); 4 Department of Chemistry and Biochemistry, University of Tulsa, Tulsa, OK 74104, USA; robert-sheaff@utulsa.edu; 5 Department of Psychology, Oklahoma State University, Stillwater, OK 74078, USA; james.grice@okstate.edu

**Keywords:** *Apis mellifera*, cloud seeding, learning, potassium iodide, silver iodide

## Abstract

Silver iodide (AgI) and potassium iodide (KI), which are used in cloud seeding, were administered to bees in a variety of pretreatments (low or high dosing) and analyzed through a series of experiments to determine the effect on bees’ ability to learn. Cloud seeding is the process of dispersing chemicals into an already-formed cloud to attract water molecules that fall to Earth as rain or snow. These chemicals then enter the ecosystem through water and soil. Honey bees were used because they represent a robust and ecologically appropriate model organism to study the behavioral impacts of cloud seeding. The first experiment utilized a shuttle box to test whether honey bees could avoid shock in a punishment experiment. Results revealed that the majority of the pretreatments did inhibit bees’ ability to learn to avoid shock. Experiment 2 consists of two proboscis extension reflex experiments (PER) where bees are trained to associate an odor with a sucrose feeding. Using the PER paradigm, we investigated simple conditioning and odor discrimination. Results revealed that in both the simple conditioning and discrimination experiments, learning was inhibited by the pretreatment of chemicals regardless of dosing amount. The final experiment explored reward discrimination in a free-flying flower patch paradigm. Results revealed that learning ability was not affected; however, return times were greatly impacted. Overall, results showed that AgI and KI throughout each experiment (i.e., shuttle box, PER, and free-flying discrimination) had some degree of negative effect on honey bee behavior.

## Simple Summary

Two of the most popular chemicals used in cloud seeding are silver iodide (AgI) and potassium iodide (KI). Despite the increasing use of cloud seeding chemicals, surprisingly little is known about their effect on behavior. Using the honey bee as a model organism because of its critical role as a pollinator, this study evaluated the impact of AgI and KI on passive avoidance, the Pavlovian conditioning of proboscis extension, and the choice behavior of bees foraging on an artificial flower patch. Individual honey bees were exposed to either high or low dosing and then tested in one of the three standard honey bee learning paradigms. The results revealed that AgI and KI affected honey bee performance in all three paradigms, with the greatest impact on the PER and the least on the flower-patch paradigm. These experiments are unique not only as they represent the first study investigating these chemicals on honey bee learning, but also because we used three different paradigms.

## 1. Introduction

Metals continue to be an environmental concern both from natural sources [1] and as byproducts of human activities such as mining, smelting, medicine, and manufacturing [2]. The range of metals is broad, and humanity’s persistent indifference to environmental impact has been a problem throughout history. Health effects of lead exposure, for example, were well known in ancient Rome [3]. Nevertheless, the many conveniences afforded by lead in everyday life led Romans to minimize the risks it presented. Lewis [3] characterizes attitudes well in saying, “Romans of yesteryear, like Americans of today, equated limited [metal] exposure …… with limited risk”. The same can be said of mercury [4,5,6], yet even today, we equate small ‘trickle’ exposures with limited risk [7,8,9]. Geoengineering represents an emerging byproduct source for environmental contamination with silver iodide and potassium iodide.

Weather geoengineering to enhance precipitation in drought regions is based on the fact that only a small fraction of the water in clouds becomes precipitation that reaches the ground [10,11,12,13]. In fact, Braham [14] found that only about 20% of the water in ordinary thunderstorm clouds ends up as ground-level precipitation. Thus, the opportunity exists to bring rain to drought areas through advances in cloud seeding technology.

Here, we investigate the effects of silver iodide (AgI) and potassium iodide (KI) on honey bee learning. The rationale behind our selection of these chemicals is that they are two of the most widely used chemicals for cloud seeding. The impetus for this study stems from the increased use of AgI and KI in weather geoengineering worldwide over the past fifty years. This increase leads to the problem of a new source of low-level environmental exposure to these compounds (e.g., [15,16,17]).

Metals are well known to affect cognitive abilities [18], which suggests that AgI and KI will impair honey bee olfactory associative learning via neurotoxicity or thyroid-analog mediated endocrine disruption. This could be manifested in several ways: Appetitive, reversal, and/or reversal learning. Thus, three different learning paradigms were used to test this hypothesis; our study uses independent test of appetitive and aversive learning (PER and shuttle-box experiments) plus examines reversal learning and has a free-flying scenario (flower patch experiment).

Honey bees are an excellent model organism for such studies because of their vital role in agriculture, diverse conditioning techniques, and accessibility to their nervous system. To our knowledge, there are no previous experiments investigating the effect of AgI and KI on honey bee learning.

The effects of silver and potassium iodide on organisms have reached differing conclusions. Some research has found that these substances negatively affect both plants and animals metabolically [19,20,21]. However, other work shows that AgI and KI are unlikely to harm organisms [22,23].

In terms of geoengineering technology, many believe that the concentration of AgI and KI are too low to affect the environment [15,20,22,24]. Indeed, ecological data of soils and streams appear to confirm that environmental concentrations are too low to have a biological effect immediately [25]. Nevertheless, long-term environmental exposure to chemical compounds in trace amounts can have biological consequences that are not identifiable in short-term studies [26,27]. For instance, potassium iodide has been shown to produce biological mutations under long-term exposure [28,29], and both potassium and silver iodide show some degree of central nervous system neurotoxicity [28,30].

For example, Chicas-Mosier compared aluminum exposure in four different subspecies of honey bees and found that aluminum exposure does not immediately affect foraging; however, it does affect overall life span, circadian adherence, and motility [31]. At the colony level, Leska and colleagues [32] discovered that the distribution of toxicants is dependent on the type of metal exposure, leading to changes in the colony’s dynamics and survival. The most impacted areas of the colony due to metal toxins were honey contamination and a decrease in brood, which overall led to lower hive health [32]. Similar results were found in stingless bees. Do Nascimento and colleagues [33] found environmental metal pollutants in the honey produced by stingless bees. This research suggested that honey should be used as a natural bioindicator of environmental metal contamination [33].

Metal toxins can have detrimental effects even at sublethal doses on bee development, learning, and memory [34]. Monchanin and colleagues sampled over 1000 honey bees from various apiaries in France near old gold mines. They compared brain, head, and body size allometry, plus the bees’ ability to learn after exposure to the metals in the mines. Bees closer to the mining sites showed more severe growth defects and deficits in learning and memory than bees located further from the mines [35]. Monchanin et al. [35] also tested the effect of chronic lead exposure on honey bee learning and found that lead decreased not only honey bee olfactory learning but also head size.

In terms of cloud seeding, both AgI and KI toxicity varies greatly among species as might be expected. Thus, a particular concentration in one environment may be lethal to the dominant species, but benign in another environment simply based on species compositions.

Terrestrial plant AgI tolerance, for example, varies widely between species, ranging from µg/L to mg/L amounts in water [36]. The freshwater microbiota is negatively affected by AgI at micromolar concentrations; green algae physiology declines at concentrations less than 12.5 μM AgI, and cyanobacteria at about 0.4 μM AgI in the environment [19]. Fish and aquatic invertebrates are affected at levels as low as 0.6 μg/L of water [37]. Terrestrial vertebrates seem to be more tolerant of dietary silver, with 2.6 mg/mL of drinking-water showing lethality in rats [38]. Australian Drinking Water Guidelines (NHMRC/NRMMC, 2004) established a concentration of 0.43 mM AgI as the threshold value of this compound in potable water for humans [39].

Potassium is essential for cell function in both plants and animals and required in plant development. Thus, large amounts of potassium are required to show toxicity, but this is not true of iodide. Rodents are negatively affected by KI doses around 0.02 g/day in their diet [40]. The acute toxicity of daphnia to potassium iodide at 48 h EC50 is 7.5 mg/L of water. For plants, low concentrations of KI are beneficial in drought situations, but above 10 µM can be harmful when water is not an issue [41].

AgI in the environment (i.e., soil, water, snow) from cloud seeding ranges from negligible to 200 times the background level [22,42]. Although elevated, these higher values are still considered to be low. Nevertheless, some localities can have up to three metric tons of AgI released into the environment per year [19,37] through low level cloud seeding.

Bioaccumulation may also be an issue leading to toxic levels even when environmental metal concentration in the soil and water is minimal. Plants move the metals from the soil into the food chain, where biomagnification results in high metal concentrations, especially in upper trophic levels [43,44]. A notable example of this phenomenon is Cesium 137 in coconuts on Bikini Atoll nearly 70 years after the last atomic bomb test [45]. Another historical example is mercury bioaccumulation at Minamata Bay, Japan [46]. These historical examples highlight the long-term effects of metal toxicity.

This series of experiments uses honey bees as a model insect to explore aversive and appetitive learning in laboratory and environmental settings after bees are pretreated with either AgI or KI. Given honey bees’ critical role in agricultural pollination [47] and the extensive body of research on their ability to learn [48,49], they represent a robust and ecologically appropriate model organism to study the behavioral impacts of cloud seeding. The first experiment investigated punishment using a shuttle box apparatus. The second experiment utilized the proboscis extension response paradigm (PER) to identify bees’ ability to associate an olfactory stimulus with an unconditioned stimulus. Finally, the third experiment employed an artificial flower patch model to evaluate the ability of free-flying bees to discriminate between two colors, reinforced by varying levels of sucrose. Together, this series of experiments aims to provide a clearer understanding of the effects of silver and potassium iodide on animal learning.

## 2. Materials and Methods

A series of experiments was performed to test whether honey bees can acquire learned behaviors after ingesting either AgI or KI at varying dosages (high or low). Both AgI and KI were obtained from Sigma-Aldrich located in Saint Louis, MO, USA (reagent grade, Prod. No. 226823 and 221945). The solvent was 1 M sucrose. No chelating agents or carriers were used; however, we sonicated the AgI in 1 M sucrose to generate particles small enough to both remain suspended in the 1 M sucrose solution throughout the experiment and maximize solute. This situation may occur in nature where both dissolved and suspended AgI readily exist in a solvent such as nectar, particularly if yeast are present as a biological agent, or pond water in which a diversity of micro-organisms are present in the water column. The experiments range from highly controlled laboratory settings to less controlled natural free-flying environments. In addition, the experiments implement a variety of conditioning techniques, including appetitive and aversive stimuli, complex and simple Pavlovian conditioning, and the influence of AgI and KI on acquisition and extinction.

Honey bees were pretreated with either silver or potassium iodide at either a high or low dosing, then evaluated using one of the three experimental designs. The KI doses used were based on those considered ‘safe’ (130 mg KI radiation emergency) for the average human (62 kg), although higher single doses seem to be acceptable to some. We then scaled the dose to a 0.1 g honey bee forager (‘safe’ dose 0.2 μg KI). In order to potentially see an effect, the low honey bee dose was 10 times the ‘safe’ level, and the high dose was 100 times the ‘safe’ level, to test a range for behavioral effects or toxicity. We carried out the same procedure for dosing AgI, but we had to use animal models to determine a ‘safe’ dose [20,50]. Rabbits fed 4.2 ppm AgI in the feed resulted in no clinical signs of toxicity [20]. We based calculations on a 1 kg rabbit raised solely on 1.5 cups pellets/day. Again, we scaled the dose to a 0.1 g honey bee forager (‘safe’ dose 0.15 μg AgI). The low dose was 10 times the ‘safe’ level, and the high dose was 100 times the ‘safe’ level, to test a range for behavioral effects or toxicity.

The first experiment employed the shuttle box apparatus to test honey bees’ ability to avoid shock. The second experiment implemented the proboscis extension response to assess honey bees’ ability to associate olfactory stimuli with an unconditioned stimulus. Additionally, the second experiment explored bees’ ability to discriminate between two olfactory stimuli. The third experiment further explored discrimination utilizing the flower patch design in a natural setting using color cues. Together, these three experiments contribute to the body of the literature on silver and potassium iodide, with a particular focus on learning efficacy.

### 2.1. Experiment 1: Aversive Conditioning Using a Shuttle Shock-Box

Subjects: Two hundred and eighty honey bees (*Apis mellifera*) foragers were collected from a laboratory feeder containing 8% sucrose by volume. Test tubes with a 25 mL capacity were used to collect the bees from the feeder that was located 50 m from the hives. Foragers from two colonies were trained to visit the feeder. Once collected, the bees were immediately transferred into a bee cage provisioned with bee-candy and water. Bees were used in the experiment on the day of capture. After bees were delivered to the lab, they were food- and water-deprived for 1 h before the experiment. Each bee was only used once, and daily testing was limited to 20 bees to control for calendar variables.

Apparatus: The apparatus is a two-compartment shuttle box. This is a standard behavioral apparatus in which the animal “shuttles” between the two compartments. The apparatus is used primarily in aversive conditioning studies. Animals are tested to see if they can learn to avoid an aversive stimulus by either moving into the no-shock compartment in response to a cue (signaled avoidance) or turning off an aversive event by moving into the no-shock compartment (punishment). The animal can postpone reintroducing the aversive event by remaining in the no-shock compartment (passive avoidance).

The shuttle box is fully automated using the Parallax Microcontroller purchased from Parallax Inc., located in Rocklin, CA, USA [51,52]. This device has demonstrated excellent results in several prior experiments (e.g., [53,54,55]). Shock intensity was set to 10 VDC and 0.05 A. The rationale behind the use of this intensity was to select a shock level that elicited a mild reaction so as not to elicit sting extension and subsequent release of alarm pheromone that might influence the results. The compartment’s internal dimensions are 140 mm × 20 mm × 10 mm. The compartments hold 55 shock grid pins and two infrared LED lights with receptors. Pins are 1 mm in diameter and are placed 2.5 mm apart. To ensure all honey bees remained connected to the grid throughout the experiment, a clear Plexiglas sheet was placed on top of the compartments. To prevent pheromone communication, separate shuttle boxes were employed and cleaned after each bee completed the experiment.

Master bees controlled when the shock was emitted based on their body placement in the apparatus. In contrast, yoke bees were shocked based on the master bee’s position. The critical difference between master and yoke bees is that the master bee can control the onset and offset of shock, and the yoke bee cannot. Although the bees receive the same number, duration, and intensity of shock. Once the master bee entered the shock side of the compartment, a continuous shock was administered until the bee moved to the no-shock side of the compartment. Once the bee entered the no-shock side of the compartment, the shock was terminated. The shuttle box was first created by Abramson et al. in 1982 and has since been used to study a variety of animals, including honey bees, cockroaches, and crickets (e.g., [56,57,58]).

Experimental Design: Subjects were separated into seven master groups (listed in Table 1), each with yoked pairs. Before each bee entered the apparatus, they were transferred from the bee box back into a 25 mL test tube with small 1 mm ventilation holes and placed into the freezer for sedation. Once sedated, bees were put into a wire-lopped harness. Once harnessed and no longer sedated, bees were pretreated with 10 μL of one of the chemical solutions presented in Table 1. After all of the pretreatment was consumed, the bees were returned to the test tube for a 115 min waiting period. This period was essential to ensure that each bee had sufficient time to metabolize the pretreatment. After the waiting period, two bees were placed at a time in the shuttle box (one master and one yoked), and a 2 min acclimation period commenced. During the acclimation period, the shock was turned off to allow the bees to roam the apparatus. Directly following the acclimation phase, the experimental phase commenced, during which the shock was administered contingent on the master bee’s position in the apparatus for a 5 min interval. For data management purposes, the proportion of time spent on the correct side (non-shock) was divided into 60 s intervals.

### 2.2. Experiment 2: Proboscis Extension Response (PER)

The proboscis extension response technique is commonly used to study learning in honey bees. This paradigm restrains bees and presents them with a pairing of a conditioned stimulus (CS), such as a floral odor, with a sucrose unconditioned stimulus (US). The US is applied briefly to the antennae, then, when the proboscis is extended, the bee is allowed to feed on the sucrose. After several CS-US presentations, the bee extends its proboscis to the CS odor before the administration of the US [57,58].

Subjects consisted of 288 honey bees (*Apis mellifera*), collected at a feeding station following the methodology of Experiment 1. Once collected, each bee was placed in a modified metal bullet casing [59] and secured by duct tape. Following containment, each bee was fed until satiated and put aside to be tested 24 h later. On the subsequent day at the designated time, the bees’ antennae were briefly touched with sucrose (but not allowed to feed) to emit the PER. If a robust PER was elicited, the bee was used. If not, the bee was discarded. This procedure limited subjects to healthy bees for experimentation. Following the health pretest, the bees that passed were set aside for 15 min before receiving their experimental pretreatment. To control for calendar variables such as changing field conditions and temperature, bees from several different experimental groups were run daily.

Procedures: Two types of PER experiments were performed.

The first (i) experiment examined whether the pretreatment of AgI or KI affected simple conditioning with the use of only one CS. Following the acquisition phase, an extinction phase was initiated in which the CS no longer followed (sucrose feeding) the US. The extinction phase was implemented to evaluate the chemical’s potential formation and persistence on the conditioned response. The rationale for including extinction was determined by the possibility that an agrochemical may not affect acquisition; however, it could affect persistence. Alternatively, the chemicals may seemingly inhibit acquisition; however, the response may recover over the extinction period. Employing an extinction procedure is seldom used in agrochemical studies of the PER and, therefore, was included [60,61,62].

In the second experiment (ii), complex Pavlovian conditioning was investigated by studying discrimination learning after ingestion of Agl or KI. In these experiments, each bee received two conditioned stimuli. The CS paired with a feeding (i.e., the US), symbolized as CS+, and the CS not paired with a feeding, symbolized as CS−. These discrimination experiments aimed to determine whether exposure to AgI or KI affects simple and complex learning differently, or at all. For example, a chemical may affect discrimination learning but leave the simple learning unaffected.

(i) Paired CS-US Experiment: Honey bees were harnessed in tubes secured by duct tape and pretreated with one of the chemical doses described in Table 2, 15 min before PER conditioning. A plastic 20 cm syringe was used to present the CS. To turn the syringe into an “odor cartridge,” a cotton ball was secured to the plunger of the syringe with an uncolored thumbtack, and 2 µL of the odor was applied to the cotton ball. Experiments were conducted in a portable fume hood purchased from the company The Airhood, located in Kwun Tong, Hong Kong (Airhood model AH-01AE), measuring 14 cm D × 22 cm W × 33.5 cm H. The rationale behind the use of the fume hood was to prevent the CS odor (and any others) from lingering in the air between trials.

Simple conditioning: Simple conditioning consists of two phases: Acquisition and extinction. Each group consisted of 24 bees who all received 12 acquisition trials in which the CS (odor) was paired with the US (sucrose feeding). Following the 12th acquisition trial, all bees received a further 12 trials but without US (i.e., presentation of the CS only) for a total of 24 trials. Two CS odors were used (cinnamon and lavender). Counterbalancing was applied for the two different odors, where 12 of the 24 bees received a CS of cinnamon and the remaining 12 received a CS of lavender. The two odors used in the simple conditioning were also used in the discrimination experiment.

Similarly to our previously published experiments (e.g., [61]), the intertrial interval was set to 10 min, in which the CS was presented for 3 s, and the US for 2 s. A non-overlap paired procedure was implemented for each CS presentation. When the US was presented, the other was terminated. The dependent variable, recorded visually, was whether the bee extended its proboscis during the CS but before the US. If the proboscis was extended during the CS, a “1” was recorded; if not, a “0” was recorded. The proboscis extension or failed extension was also recorded when the US was presented. As no US was present during the extinction period, we only recorded the presence or absence of a response during the CS. The US was applied using an unscented wooden toothpick. The end of the toothpick was dipped into a 50% (by weight) sucrose solution. Following the CS presentation, the sucrose-dipped toothpick was lightly touched to one of the antennae, causing the proboscis to extend. They were allowed to consume the sucrose on the toothpick for 2 s. Unlike our previously published PER experiments, the second set employed a within-subject discrimination control rather than unpaired controls.

(ii) Discrimination PER Experiment: As in the previous experiment, the CS odors were cinnamon and lavender, the CS duration was 3 s, and the US duration was 2 s. A non-overlap procedure was used where the CS was terminated before the US was presented. Each bee received 24 trials consisting of 12 CS+ trials and 12 CS− trials. A total of 144 subjects were pretreated following the methods of the previous experiment. The dependent variables mirrored the simple experiment, i.e., whether they extended or did not extend their proboscis to the CS+, CS−, and US. Counterbalancing was conducted with 12 subjects receiving a CS+ of cinnamon and a CS− of lavender; the remaining 12 received a CS+ of lavender and a CS− of cinnamon, ensuring equal assignment across the 24 animals per group. To randomize the CS+ and CS− presentations, a pseudorandom sequence of ABBABAABABBABAABABBABAAB was initiated with “A” always beginning with a CS+ and the “B” representing the CS−.

A major difference between the two experiments is the intertrial interval. In the simple experiment, the intertrial interval was 10 min. In the discrimination experiments, it was reduced to 5 min. The rationale behind this modification is that a 10 min intertrial interval in the discrimination experiment would lead to a 20 min overall intertrial interval between both CS+ presentations, instead of the intended 10 min interval. Therefore, to keep intertrial intervals consistent, the discrimination experiment administered a 5 min intertrial interval.

### 2.3. Experiment 3: Artificial Flower Patch

Free-flying honey bees were trained to a feeding station 30 m from the hive. An artificial flower patch replaced the Petri dish at the feeding site for an experiment. Each trial of an experiment utilized a new set of uncaged, free-flying naive honey bees (*Apis mellifera*) that had no previous experience with the artificial flower patch. The bees were trained to the flower patch following the methods of Wells et al. (e.g., [63,64,65]). Four or fewer bees were used in each trial of the experiment, each uniquely marked with Testor’s (Testor Corporation, Rockford, IL, USA) enamel paint. Any additional bees that visited the flower patch were removed from the system. Due to differing return times, there were only one or two bees on the flower patch at a time, which mimicked a natural foraging environment.

Flower Patch: All experiments used free-flying honey bees (*Apis mellifera ligustica*) who foraged outdoors on artificial flower patches consisting of 36 flowers. Each flower was equally spaced 75 mm apart in rows and columns of a 6 × 6 grid, following the Cartesian coordinate system on a brown pegboard, according to the design of Wells et al. (e.g., [66,67,68,69,70]). Flowers consisted of a 28 mm × 28 mm Plexiglas square, 6 mm thick, with a 5 mm diameter, and a 4 mm deep well in the center that held the sucrose reward. Each flower was mounted on a 90 mm pedicel of 5 mm dowelling.

Honey bees had a choice between blue or white flowers in the flower patch apparatus. Flower patches consisted of flowers of equal numbers, randomly arranged based on color (blue or white) within the array. Flower color was created by painting the lower surface of each flower blue or white with enamel paint (Testors^TM^ paint Nos. 1208 blue, 1245 white). The reflectance spectra for the paints, and a color hexagon that depicted how honey bees perceive these colors, can be found in Hill et al. [71]. Flowers were washed in unscented detergent and then triple rinsed and allowed to dry after each use. The flower patch method has previously been used to determine flower color preference, reward molarity discrimination, and reward quantity discrimination [64,72,73]. This method was used here to determine whether AgI or KI affects learning to associate flower color with reward quality.

Experimental Design: The experiment had three treatment conditions given to each bee. Treatment 1 offered foragers 8 μL of 1 M sucrose in both blue and white flowers. Its purpose was to gather baseline data on color choice before bees were given a situation where rewards differed between flower colors. Treatment 2 presented bees with an energy-maximization learning experience. It offered foragers the choice of 8 μL of 1.5 M in blue flowers versus 8 μL of 0.5 M sucrose in white flowers. Treatment 3 offered foragers the choice of 8 μL of 1.5 M in white flowers versus 8 μL of 0.5 M sucrose in blue flowers. Approximately half of the bees were given Treatment 3 before Treatment 2 to control for flower color preference, initial appetitive learning, and appetitive reversal learning (e.g., [74]). The experiment had 5 dose groups (see Table 3).

Each bee was given a single dose of 10 μL 1 M sucrose containing either AgI or KI upon return to the flower patch from the hive immediately after landing on the first flower visited (Table 3). After feeding bees were held for 20 min and then released. Initial return time was recorded using a digital stopwatch (with split functions) running continuously. Treatment 1 conditions prevailed until all bees returned from the hive after being released. The color of each flower visited by a bee was recorded for each treatment of an experiment.

## 3. Statistical Analysis

Observation Oriented Modeling (OOM) https://idiogrid.com/OOM/index.php/77-2/?succes=1762815808 (accessed on 21 October 2023) software was used to analyze all experimental data. OOM allows researchers to analyze data based on patterns within the observations or compare a given set of observations to a predicted pattern based on a priori hypotheses [75,76]. Unlike traditional statistical analyses, OOM is not bound to assumptions of normality, random sampling, or homogeneity of variances, making it ideal for small sample sizes and behavioral data. OOM is centered around patterns that can be identified in visual plots of the observations. Once the data is graphically observed and analyzed, OOM produces a Percent Correct Classifications (PCC) statistic that tells the researcher exactly how many observations or individuals fit the expected pattern [77]. This statistic is much like an effect size, but one that is readily interpretable by laypersons and professionals alike. Further, instead of *p*-values, OOM provides *c*-values, which relate to the PCC by determining if values equal to or greater than the observed PCC can be randomly produced (or haphazardly produced). We have used OOM in several previous publications (e.g., [78,79,80]) and found it useful.

## 4. Results

### 4.1. Experiment 1: Aversive Conditioning Using a Shuttle Box

Data from all 280 honey bees were analyzed in OOM to determine whether silver or potassium iodide impaired bees’ ability to avoid shock. Two concatenated pattern analyses were conducted: one for master bees and one for yoked bees. The two analyses were used to compare the seven different groups’ quantity of time (in seconds) spent on either the correct or incorrect side. Based on the prior literature, we expected the bees to spend most of their time on the no-shock side of the apparatus, demonstrating their learning ability. However, if this pattern does not hold, it would support the hypothesis that chemical exposure impairs honey bee learning. Therefore, all bees in all groups were expected to stay on the correct side (the no-shock side) more than on the incorrect side of the apparatus. The percent correct classifications (PCC) indices represent the numbers of observations or individual bees’ pattern of responses that are consistent with this expectation during the 5 min trials. In each of the figures below seven trials are included with the first two representing the habituation phase and the following five the experimental phase.

This study design included three control groups: one group of bees that experienced shock and were fed a 50% sucrose solution before entering the shuttle box, while the other two groups did not receive shock during the 5 min experimental trials. Among these latter two groups, one was fed a 50% sucrose solution before entering the apparatus, and the other was not.

In order to show learning, bees were expected to stay on the correct side more than the incorrect side per trial (60 s per trial) as shown by spending 30 s or more on the correct side (above 50%). If bees spent approximately 30 s on each side throughout the trials, their behavior would suggest that they were unable to learn to avoid shock. The yoked bees did not have the ability to learn because they were shocked when the master bees were shocked and not based on position.

**Figure 1:**
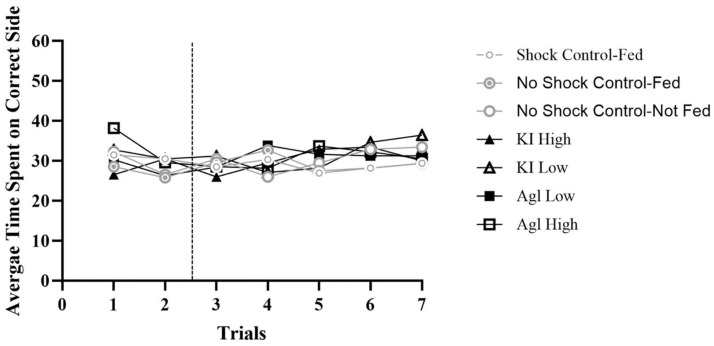
Averages across all yoked groups. *Note.* The *y*-axis represents the 60 s possible for each trial and the *x*-axis represents each trial. The hashed vertical line is used to separate the two habituation trials from the five experimental trials. All yoked groups resulted in roughly 30 s on the correct side showing 50% of their time was spent on the correct side.

Therefore, the yoked pairs for all groups spent about 50% of their time on the correct side of the shuttle box (overall PCC Range = 47–54%, c-value range = 0.24–0.69). These bees were shocked when the master bees were shocked and therefore did not have a side to learn to avoid. Consequently, they spent a similar amount of time (roughly 50%) on the correct side as the control bees who were not shocked (see Figure 1). These results indicate that when the bees cannot learn to avoid shock, they shuttle just as the control bees who were not shocked, demonstrating that the chemicals are not inhibiting their ability to move within the shuttle box.

The control group that received shock and was fed a 50% sucrose solution before entering the shuttle box performed the best out of all seven groupings. Across all 20 bees and five trials, 85% of the 100 observations matched expectation (PCC = 85.0%, c-value < 0.0001). With regard to individual bees, each stayed on the correct side longer than the incorrect side for a majority of the trials (at least 60%), and 9 out of 20 bees stayed on the correct side longer for all five trials. These bees served as the comparison group and represented how experimental bees should perform if the chemicals did not affect learning.

Among the control bees that were not shocked, the group that was fed a 50% sucrose solution resulted in an overall PCC of 41.0% and a c-value of 0.97. The group that was not fed had a PCC of 57.0% and a c-value of 0.10. Bees in these groups moved about the apparatus with no overall preference. While some bees tended to stay on one side, others stayed on the opposite side or spent roughly equal amounts of time on either side of the apparatus. Both of these groups, then, were unable to learn how to avoid shock because the shock was not on. This finding is further highlighted by the yoked bees performing similarly to the master bees (fed PCC = 48%, c-value = 0.76; not fed PCC = 48%, c-value = 0.69).

Of the experimental groups, the highest-performing was that pretreated with silver iodide at a low concentration (overall PCC = 82.0%, c-value < 0.0001). The high PCC indicates that this group, in the aggregate, learned to identify which side of the shuttle box did not produce shock. With regard to individual bees, 16 stayed on the correct side longer than the incorrect side for a majority of the trials (at least 60%), and 11 out of 20 bees stayed on the correct side longer for every trial. However, there were 4 bees that spent more time on the correct side of the apparatus for a minority of trials (individual PCCs = 40%; viz., 2 of 5 trials). The next highest-performing group was the high concentration of potassium iodide (overall PCC = 72.0%, c-value < 0.0001), with 16 individual bees staying on the correct side longer than the incorrect side for a majority of the trials (at least 60%), and 8 out of 20 bees staying on the correct side longer for every trial. Again, only 4 bees spent more time on the correct side of the apparatus for a minority of trials (individual PCCs ≤ 40%). The overall PCC for the highest concentration of silver iodide bees was only 65% (c-value = 0.004), with 15 individual bees staying on the correct side longer than the incorrect side for a majority of the trials (at least 60%), and 6 out of 20 bees staying on the correct side longer for every trial. In all, 5 bees in this group spent more time on the correct side of the apparatus for a minority of trials (individual PCCs ≤ 40%). The low concentration of potassium iodide bees performed the worst, overall (PCC = 64%, c-value = 0.02). A total of 15 of the individual bees stayed on the correct side longer than the incorrect side for a majority of the trials (at least 60%), and only 3 out of 20 bees stayed on the correct side longer for every trial. Altogether, 5 bees in this group spent more time on the correct side of the apparatus for a minority of trials (individual PCCs ≤ 40%).

**Figure 2:**
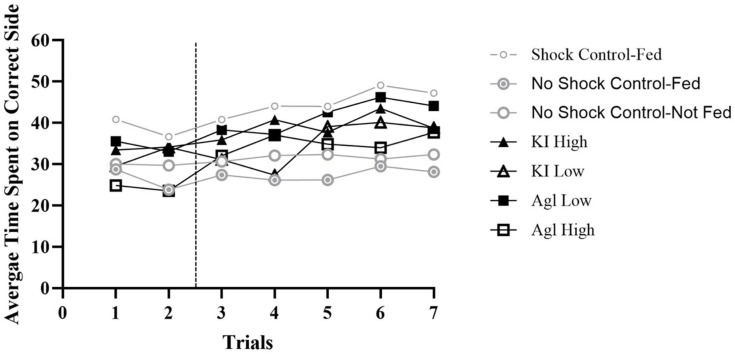
Averages across all master groups. *Note.* The *y*-axis represents the 60 s possible for each trial and the *x*-axis represents each trial. The hashed vertical line is used to separate the two habituation trials from the five experimental trials. The majority of master bees spent more than 30 s (above 50%) on the correct side of the shuttle box.

In summary, the bees’ ability to avoid shock was inhibited by most of the chemical concentrations (see Figure 2). The groups that were least inhibited by the chemical concentrations were the low concentration of silver iodide (82%) and the high concentration of potassium iodide (72%). Conversely, the concentrations that most affected the bees’ ability to learn to avoid shock were the high concentration of silver iodide (65%) and the low concentration of potassium iodide (64%). These results provide insight into how different concentrations of both chemicals influence honey bee behavior in a punishment experiment and contribute to the debate on the effects of cloud seeding on animal behavior.

### 4.2. Experiment 2: Proboscis Extension Response (PER)

Paired CS-US Experiment: A total of 144 bees were analyzed across 24 trials, divided into 12 acquisition trials and 12 extinction trials for analysis. A concatenated pattern analysis was conducted to determine whether bees responded correctly or incorrectly across all 12 trials, as shown by honey bees extending their proboscis. OOM revealed a PCC for each bee individually by computing the percentage of ‘correct’ trials (i.e., summing the correct trials, dividing by 12, and multiplying by 100). The software also computed a total PCC. The total PCC is calculated by summing the total number of correct trials across all bees and dividing by the total number of trials across all bees and then converting that proportion into a percentage.

*Control Groups.* The control groups consisted of two subsets: bees pre-treated with sucrose and bees who were not pre-treated. During the acquisition phase, the non-pre-treated group correctly responded to the odor 64.93% of the time (c-value < 0.0001). Individually, 15 out of 24 bees responded correctly at 60% or above over 12 trials. The pre-treated control bees correctly responded to the odor 65.97% of the time (c-value < 0.0001). Individually, 14 out of 24 bees achieved an accuracy of 60% or above throughout the 12 acquisition trials.

In the extinction phase, the pre-treated control group continued to extend their proboscis 52.8% of the time (c-value = 0.27), with 11 out of 24 bees maintaining this behavior throughout the 12 extinction trials at 60% or above. The control group that was not pre-treated exhibited a proboscis extension 47.22% of the time (c-value = 0.84), with 8 out of 24 bees continuing this behavior at 60% or above.

*Experimental Groups.* There were four experimental groups consisting of high and low concentrations of silver and potassium iodide. All groups extended their proboscis correctly less than 15% of the time during both the acquisition and extinction phases. These results indicate that bees fed, either concentrations of silver or potassium iodide were not able to be classically conditioned.

The group of bees fed the high concentration of potassium iodide correctly responded 9.03% of the time (c-value = 1.00). In this group, 18 out of 24 bees did not respond correctly at all over the 12 acquisition trials (i.e., individual PCCs = 0%). During extinction, this group responded with decreased correct responses, with only 5.56% of the bees extending their proboscis (c-value = 1.00). The group of bees fed the low concentration of potassium iodide responded correctly during acquisition 5.90% of the time (c-value = 1.00). In total, 17 out of 24 bees did not respond correctly at all over the 12 acquisition trials. Unexpectedly, the PCC increased to 7.76% during extinction (c-value = 1.00), possibly due to spontaneous recovery or random extension.

The group of bees fed the high concentration of silver iodide had 12.85% correct responses during acquisition (c-value = 1.00), while the low concentration group had 5.56% (c-value = 1.00). In the high concentration group, 18 bees never responded correctly, while in the low concentration group, 15 out of 24 bees never responded correctly. During extinction, the high concentration group decreased to 9.72% correct responses over the 12 trials (c-value = 1.00), and the low concentration group decreased to 4.86% (c-value = 1.00).

**Figure 3:**
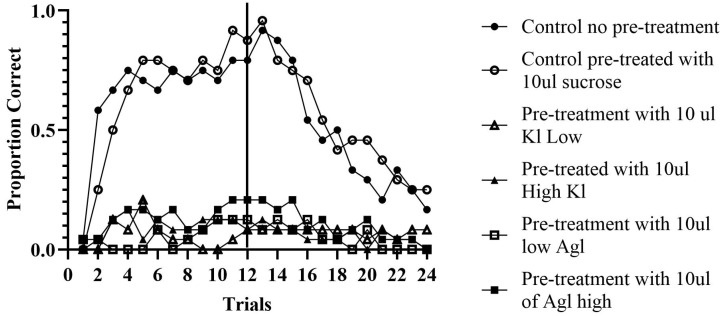
PER paired experiment. *Note.* The first 12 trials represent acquisition, and the next 12 trials extinction where the U.S. is omitted. The results show that the ability to associate an odor with a sucrose feeding is severely retarded by preexposure to KI or AgI. In contrast, the proportion of control bees given no pretreatment or pretreatment with a sucrose feeding before PER conditioning, readily acquired the association between odor and feeding.

These results indicate that the chemicals drastically inhibited the honey bees’ ability to learn to associate food with odor (see Figure 3). The control groups had correct responses approximately 65% of the time during acquisition, which decreased to around 50% during extinction. However, none of the experimental groups exceeded 15% correct responses in either acquisition or extinction. These findings suggest that silver and potassium iodide impact honey bees’ ability to be classically conditioned, highlighting the potential effects of cloud seeding chemicals on animal behavior.

### 4.3. Discrimination PER Experiment

A total of 144 bees were analyzed across 24 trials. Utilizing the ABBABAAB pattern, correct responses, as indicated by proboscis extinction for the odor previously paired with sucrose indicated by A and the odor not previously paired with sucrose indicated by B, an incorrect response was used to determine the pattern for analysis.

*Control Groups.* The control groups consisted of a condition that was pre-treated with sucrose and a condition that was not pre-treated. The condition that was not fed fit the expected pattern 80.03% of the time (c-value < 0.0001). Out of this group, 20 out of 24 bees correctly discriminated between the two odors. Similarly, the pre-fed condition fit the expected pattern 77.60% of the time (c-value < 0.0001). In this group, 18 out of 24 bees correctly discriminated between the two odors. These results reveal that the bees did classically condition, and pre-treating and not pretreating the bees did not make a substantial difference in the behavior of the bees.

**Figure 4:**
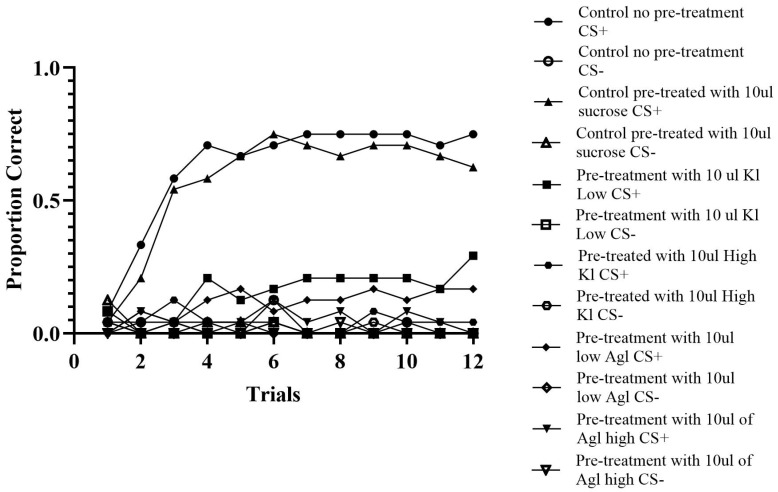
Discrimination. *Note:* The results of the discrimination experiment where each bee is given two odors, one of which is paired with a sucrose feeding (CS+) and the other is not (CS−). Consistent with our previous PER experiment (experiment 2), the results show that the ability to discriminate between two odors is severely retarded by preexposure to KI or AgI. In contrast, the proportion of control bees given no pretreatment or pretreated with sucrose feeding readily discriminated between the CS+ and CS−.

*Experimental Groups.* The varied concentrations of silver and potassium iodide influenced all experimental conditions. As anticipated, the low concentrations were the least impacted, while the high concentrations showed the greatest deficits. The lowest concentration of potassium iodide aligned with the pattern 57.29% of the time (c-value = 0.0001). Individually, 20 out of 24 honey bees did not display results exceeding 60%. Additionally, there was roughly a 20% decrease from the least affected experimental condition compared to the controls, clearly indicating that the chemicals are impacting the honey bees’ ability to discriminate (see Figure 4). Similarly, the condition, comprising bees fed the lowest concentration of silver iodide, responded to the correct odor 55.38% of the time (c-value = 0.005). There was a total of 21 out of 24 bees that did not respond correctly 60% of the time.

The high concentrations of silver and potassium iodide resulted in the largest learning deficit, see Figure 4. The high potassium iodide concentration fits the pattern at 51.22% in total (c-value = 0.30), not providing evidence for learning. Zero individuals in this group responded over 60% of the time. Likewise, the high silver iodide concentration fit the pattern by extending their proboscis to the pre-treated odor only 52.08% of the time (c-value = 0.17). These results indicate that the bees were not able to associate a specific odor with reward and therefore were not able to discriminate between the two odors when consuming the chemicals at either concentration.

### 4.4. Experiment 3: Artificial Flower Patch

**Figure 5:**
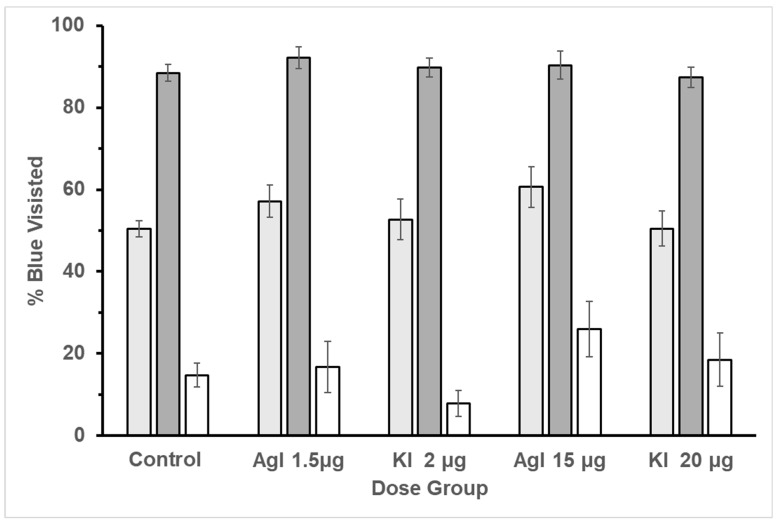
Flower visitation for each dose. The group of three bars for each dose represents the three treatments (bar = mean + se). Treatment 1 in each group is the light gray bar; both blue and white flowers offering 1 M sucrose. Treatment 2 in each group is the dark gray bar; blue flowers with 1.5 M sucrose and white flowers with 0.5 M sucrose. Treatment 3 in each group is the white bar; blue flowers with 0.5 M sucrose and white flowers with 1.5 M sucrose. Treatment 1 always came first. Treatment 3 preceded Treatment 2 half of the time.

A concatenated ordinal pattern analysis was utilized to test the correct choice for each of the five groups over the trials. The percentage of blue flower visits was used to determine the correct choice. The pattern was determined by collecting the percentage of blue visits in each of the treatments. In treatment two, the percentage of blue visits had a higher concentration of sucrose and therefore should have more visits than the white flowers, and the opposite for treatment three. All groups fit the expected pattern, resulting in PCC of 100% and c-values equal to or less than 0.001. These results indicate that bees were still able to learn to discriminate reward in a free-flying experiment (Figure 5).

**Figure 6:**
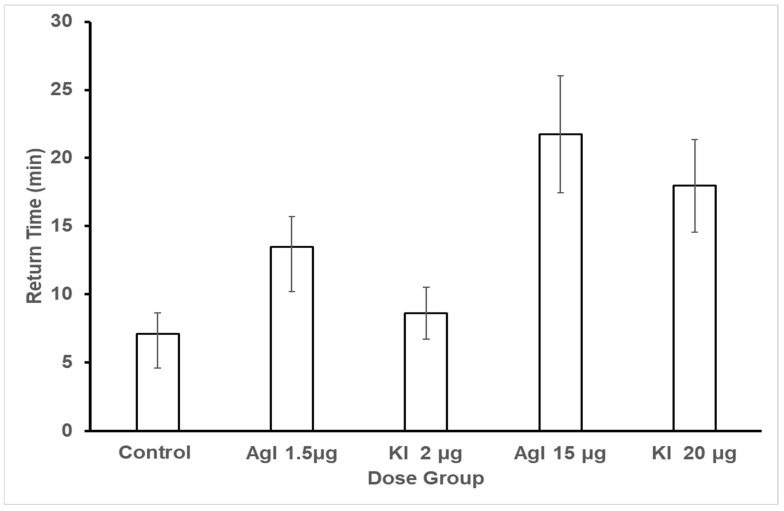
Return time initially after dosing. *Note*. Each bee received one of the five treatments (categories on the *x*-axis) after establishing crop attachment to the feeding station. Each bee was dosed once (treatment) with AgI or KI and immediately caged for 20 min. The bees’ return to the flower patch to resume foraging was then recorded. Both the high dose of AgI and KI significantly lengthened the time to return to foraging when compared to the control (1 M sucrose without AgI or KI). The low dose of AgI but not KI also significantly increased return time, but not to the degree of the high dose.

However, return times were affected by treatment with silver or potassium iodide. Return time data were analyzed with a crossed orderings ordinal analysis in the Observation Oriented Modeling software [75] using the observed means (see Figure 6) to define the ordinal pattern to be tested. Results indicated that bees in the control group did have shorter return times than three of the experimental groups: Agl low (PCC = 76.96%, c-value = 0.01), Agl high (PCC = 65.10%, c-value = 0.07), and KI high (PCC = 72.78%, c-value = 0.02). The control bees return times were not distinguishable from those of the KI low bees (PCC = 48.96%, c-value = 0.48). With regard to the experimental groups (see Figure 6), the low Agl bees had longer return times than the low KI bees (PCC = 71.32, c-value = 0.02), the high KI bees had longer return times than the low KI bees (PCC = 69.58, c-value = 0.02), and the high Agl bees had longer return times than the low KI bees (PCC = 62.11, c-value = 0.09). These results revealed an impact on return times between the five groups.

## 5. Discussion

Initially, weather geoengineering was limited to time and places where droughts were in progress. This approach has produced marginal results, which may not be surprising in hindsight. Clouds in the drought regions tend to be less frequent and carry less water, creating a losing scenario for cloud seeding [81]. An alternative approach where cloud seeding continues in non-drought conditions has proven more successful [81]. Still, while reducing the severity of drought conditions, this approach increases the byproducts AgI and KI in the environment.

The experiments reported here are unique in that, to our knowledge, this is the first time the chemicals AgI and KI used in cloud seeding have been used to investigate whether they influence honey bee learning. As we had little previous literature to consult, we opted for a “shotgun” approach where we used three different experimental designs and employed both appetitive and aversive stimuli.

Silver Iodide. There are multiple pathways where chronic exposure to silver negatively impacts organisms (see reviews: [82,83]). These include binding to DNA that causes DNA condensation which interferes with genome replication. Also, gene activation by Ag has also been reported leading to a chain of events culminating in cell apoptosis. In terms of cellular physiology, silver interacts with numerous proteins with the consequence that conformational changes occur. This is particularly noticeable in mitochondrial electron transport chain activity, decreasing ATP production. Finally, silver interacts with cell receptors. Nevertheless, silver in the form of AgI exhibits low solubility in water, which is the basis for the argument that the potential for adverse effects caused by AgI in the environment is minimal. Countering that argument is data that actually show AgI decreased photosynthesis and respiration in soil bacteria, in both aquatic green algae and cyanobacteria [19]. Moderate cellular toxicity was also reported. Medically, silver iodide has been linked to health risks that include toxicity, reproductive disorders, developmental defects, and cancer [84].

Once ingested, the amount of time AgL takes to pass through the gastrointestinal tract is associated with the amount of metal absorbed; however, there are species differences [85]. High levels of Agl can cause nausea, vomiting, diarrhea, skin irritation, eye irritation, respiratory problems, headache, and argyria in humans [19]. Silver iodide ingestion also results in transcription of the sodium/iodide sym-porter (NIS) gene and of the phytoene desaturase (PDS) gene, which can lead to increased mutation rates in mammals [28]. In adults, AgI causes an increase in blood pressure, affecting overall body function [28]. However, previous research with AgL has divergent findings. Some research suggests that AgI is highly toxic to microorganisms and fish [22]. However, others found AgI to be a minor irritant to body tissue and, therefore, is not likely to show noticeable overt effects [50]. As is well known in pharmacokinetics, changes in formulation of the same drug can have dramatic effects on bioavailability; this may to some extent explain the divergent effects reported. In the AgI studies, difference in formulation may even be due to the environment of the organism.

Silver nanoparticles (AgNPs), however, do affect the brain in several ways [30,86,87]. First, AgNPs can instigate cell damage or death, inducing oxidative stress to neurons. Second, they can enhance inflammation, which can further contribute to neuronal damage. Third, energy production and cell death can also be affected by mitochondrial dysfunction because of silver nanoparticles. Finally, the combination of oxidative stress with inflammation can accelerate apoptosis.

We would like to note that progressive bioaccumulation of silver is well documented in crop plants and fungi with long-term exposure [82]. In marine systems, algae readily uptake the metal from which it progresses into sediment filter-feeders and fish [82]. However, AgI has low solubility and thus should not provide a source of silver for bioaccumulation [88]. However, Fajardo et al. [19] demonstrated harm from bioaccumulation for aquatic life. This may be a situation similar to minute quantities of metal mercury in industrial waste that was considered environmentally unimportant due to its insoluble nature in water, only later to discover biomodification to methyl mercury.

Potassium Iodide: According to the US Food and Drug Administration [89], KI ingestion can lead to iodism. In animals, when KI is consumed in large quantities, there is a sizable decrease in NIS and PDS mRNA (sodium/iodide symporter mRNA), which leads to increased mutation rates [28,29]. Potassium iodide also has developmental toxicity effects exhibited as impaired thyroid functions, weight change, and overall brain function and body development [26,28,40].

Potassium iodide can affect thyroid physiology in vertebrates [75]; we propose thyroid-analog mediated pathways as a possible mechanism, but this study did not measure endocrine endpoints. The physiological effects can come either from thyroid hormone regulation or thyroid blocking in vertebrates. Both issues directly impact brain development and functioning [90]. Similar hormone-producing organs are found in insects called the corpora allata and prothoracic glands. These processes regulate growth, development, and other physiological processes [91].

Iodine is an essential micronutrient for animals. Potassium iodide primarily affects the brain through the thyroid gland, specifically thyroxine (T4) and triiodothyronine (T3). However, when the thyroid gland is exposed to high amounts of iodide, the synthesis and release of T3 and T4 are temporarily inhibited through the Wolff–Chaikoff effect [92]. Like animals, iodine is considered a beneficial micronutrient for plants. However, excessive iodine inhibits growth and can have accelerate senescence, more so in terrestrial than marine flora [93,94].

Honey bee: The findings presented here highlight the issue of dispersing AgI and KI into the environment through cloud seeding. Results found that AgI and KI in all experiments (i.e., shuttle box, PER, and free-flying discrimination) had some degree of adverse effect on honey bee behavior. However, to establish generalizability of these findings, replication remains essential.

The shuttle box experiment concluded that a low concentration of potassium iodide had the largest effect on honey bees’ ability to learn to avoid shock. These bees spent only 64% of the trials on the correct side compared to 85% for the shock control bees. Each of the concentrations for both chemicals resulted in behavioral shortages.

Similarly, the proboscis extension reflex experiment revealed that all concentrations significantly affected honey bee behavior. After consuming pre-treatment sucrose laced with AgI or KI, experimental bees would not consume the sucrose reward to create the association. Furthermore, the discrimination experiment, like the shuttle box experiment, revealed that the high concentrations of chemicals had the most detrimental effects on honey bee behavior. Bees in the high concentration AgI and KI conditions only responded correctly around 50% of the time.

However, the flower patch experiment concluded that neither AgI nor KI had an effect during the outdoor free-flying discrimination experiment once they returned to the flower patch after dosing; however, AgI and KI did affect initial return times. Bees in the high silver iodide treatment had the longest return times, followed by the group receiving the high potassium iodide dose, and then the low potassium iodide and silver iodide concentration in comparison to the controls. Even though the ability to learn was consistent across all groups in the free-flying discrimination task, evaluating the extended return times does align with behavior delays due to the consumption of each chemical concentration. The differences observed between the laboratory studies and the flower patch experiment may be due to the fact that the flower patch bees did not fast before the experiment and also have the ability to unload their crop upon release at the hive.

Slowing of return time decreases daily nectar and pollen harvest by foragers. On the colony level this probably has little impact in spring and summer but rather impacts overwinter survival which is dependent on honey stores. The consequences could lead to a dearth of pollinators because one colony death results in the removal of thousands of foragers from the environment, producing a decline in plant reproductive potential.

The decline in honey bee populations [95,96,97] underscores the importance of understanding the potential side effects of introducing AgI and KI into the environment. As crucial pollinators in agriculture and food production, the health of bees is vital. Investigating the behavioral effects of iodide consumption is essential to assess any negative impacts on their foraging and ability to adapt to their environment after exposure to these chemicals.

## Figures and Tables

**Table 1:** Chemical dosings for each of the shuttle box experimental groups. Pretreatment doses 1 to 6 were all given in 10 μL 1 M sucrose. Each bee consumed the entire pretreatment.

Dose Group	Pretreatment Dose
1: AgI high	15 μg Agl/bee + shock
2: AgI low	1.5 μg Agl/bee + shock
3: KI high	20 μg KI/bee + shock
4: KI low	2 μg KI/bee + shock
5: Control I	(0 μg KI + 0 μg AgI)/bee + shock
6: Control II	(0 μg KI + 0 μg AgI)/bee + no shock
7: Control III	no pretreatment, no shock

*Note.* A total of 40 bees per dosing group (*n* = 20, master bees; *n* = 20, yoke bees) were included for each of the seven groups (*N* = 280). Each housing a master and yoked bee, in which half of the bees were shocked on the right half of the apparatus (*n* = 10) and the other half were shocked on the left half of the apparatus (*n* = 10), for counterbalancing purposes.

**Table 2:** AgI and KI groups for PER experiments. Pretreatment doses 1 to 5 were all given in 10 μL 1 M sucrose. Each bee consumed the entire pretreatment.

Group	Pretreatment Dose Group
1: AgI high	15 μg/Agl
2: AgI low	1.5 μg/Agl
3: KI high	20 μg/KI
4: KI low	2 μg/KI
5: Control I	(0 μg KI + 0 μg AgI)/bee
6: Control II	No Pretreatment

*Note.* The six groups listed were used in the paired experiment (*n* = 144), and a separate set of six groups were used in the discrimination experiment (*n* = 144), totaling 12 groupings between the two experiments. There were 24 bees in each group for a total of 288 bees between both experiments. Out of the 24 bees, 12 have a CS+ of lavender and a CS− of cinnamon, and the other 12 have the reverse of a CS+ of cinnamon and CS− of lavender.

**Table 3:** AgI and KI groups for flower patch experiments. Pretreatment doses 1 to 5 were all given in 10 μL 1 M sucrose. Each bee consumed the entire pretreatment.

Dose Group	Dose
1: Control	(0 μg KI + 0 μg AgI)/bee
2: AgI low	1.5 μg Agl/bee
3: KI low	2 μg KI/bee
4: AgI high	15 μg Agl/bee
5: KI high	20 μg KI/bee

## Data Availability

The data are available upon request.
